# Interferometric diffuse optics: recent advances and future outlook

**DOI:** 10.1117/1.NPh.10.1.013502

**Published:** 2022-10-22

**Authors:** Wenjun Zhou, Mingjun Zhao, Vivek J. Srinivasan

**Affiliations:** a China Jiliang University, College of Optical and Electronic Technology, Hangzhou, China; b University of California Davis, Department of Biomedical Engineering, Davis, California, United States; c New York University Langone Health, Department of Radiology, New York, New York, United States; d New York University Langone Health, Department of Ophthalmology, New York, New York, United States; e New York University Langone Health, Tech4Health Institute, New York, New York, United States

**Keywords:** interferometric detection, diffuse correlation spectroscopy/diffusing wave spectroscopy, blood flow index, speckle, coherence, human brain, cerebral blood flow

## Abstract

The field of diffuse optics has provided a rich set of neurophotonic tools to measure the human brain noninvasively. Interferometric detection is a recent, exciting methodological development in this field. The approach is especially promising for the measurement of diffuse fluctuation signals related to blood flow. Benefitting from inexpensive sensor arrays, the interferometric approach has already dramatically improved throughput, enabling the measurement of brain blood flow faster and deeper. The interferometric approach can also achieve time-of-flight resolution, improving the accuracy of acquired signals. We provide a historical perspective and summary of recent work in the nascent area of interferometric diffuse optics. We predict that the convergence of interferometric technology with existing economies of scale will propel many advances in the years to come.

## 1 Introduction

**Fig. 1:**
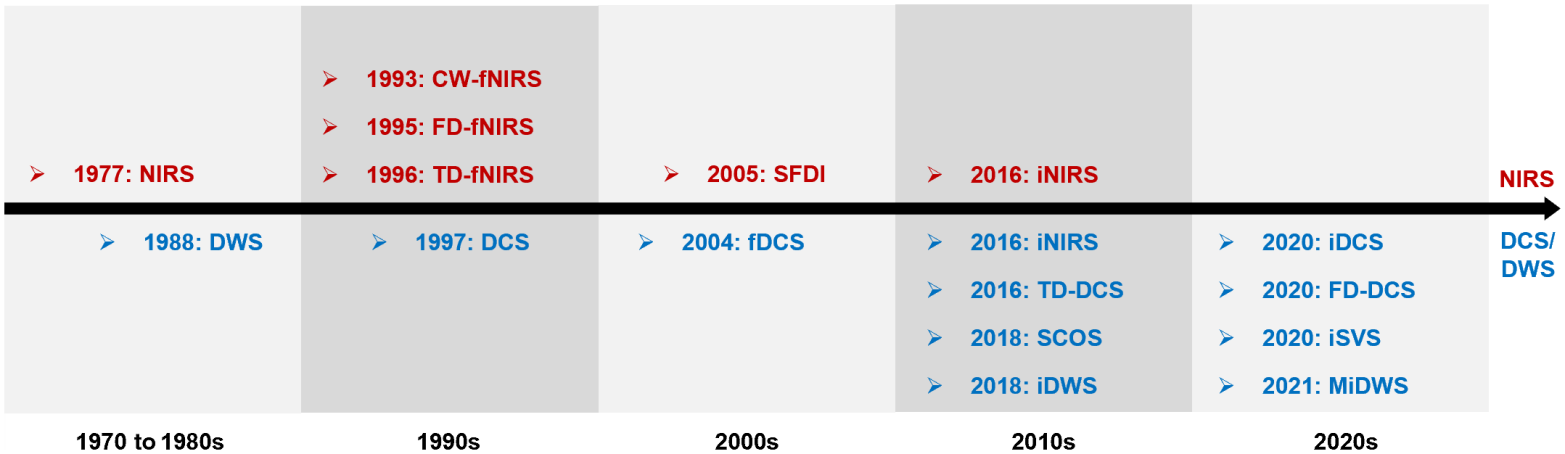
Timeline of representative advances in NIRS^2^ (red, top), DCS/DWS (blue, bottom), and related methods (iNIRS shares characteristics with NIRS and DCS/DWS, appearing in both colors). fNIRS: functional NIRS; SFDI: spatial frequency domain imaging;^7^ fDCS: functional DCS;^8^ iNIRS: interferometric NIRS;^9^ TD-DCS: time domain DCS;^10^ SCOS: speckle contrast optical spectroscopy (for human brain^11^); iDWS: interferometric DWS;^12^ FD-DCS: Fourier domain DCS with heterodyne holographic detection;^13^ iSVS: interferometric speckle visibility spectroscopy;^14^ and MiDWS: multiexposure iDWS.^15^ A growing consensus is emerging on the insertion of the letter “i” before a technique to denote “interferometric.”

Diffuse optics seeks to characterize and image biological tissue with multiply scattered near-infrared light. From the inception of this scientific field, the potential of near-infrared light to probe brain physiology was recognized.^1^ Diffuse optics is divided according to the measured signals, with light absorption and scattering for near-infrared spectroscopy (NIRS)^2^ or coherent light fluctuations (CLFs) for diffuse correlation spectroscopy (DCS),^3^ and according to detection methodology, with continuous wave (CW),^4^ time domain (TD),^5^ frequency domain (FD),^6^ or spatial frequency domain.^7^ CW NIRS was first demonstrated around 45 years ago (Fig. 1)^1^ and remains widely used for functional brain measurements.^16^^,^^17^ In the 1980s, researchers recognized the inherent ambiguities of CW measurements and explored novel dimensions, by resolving tissue responses in the TD^18^ and FD.^19^ These measurements are now considered to be quantitative benchmarks to disambiguate absorption and scattering and to achieve depth sensitivity.

**Fig. 2:**
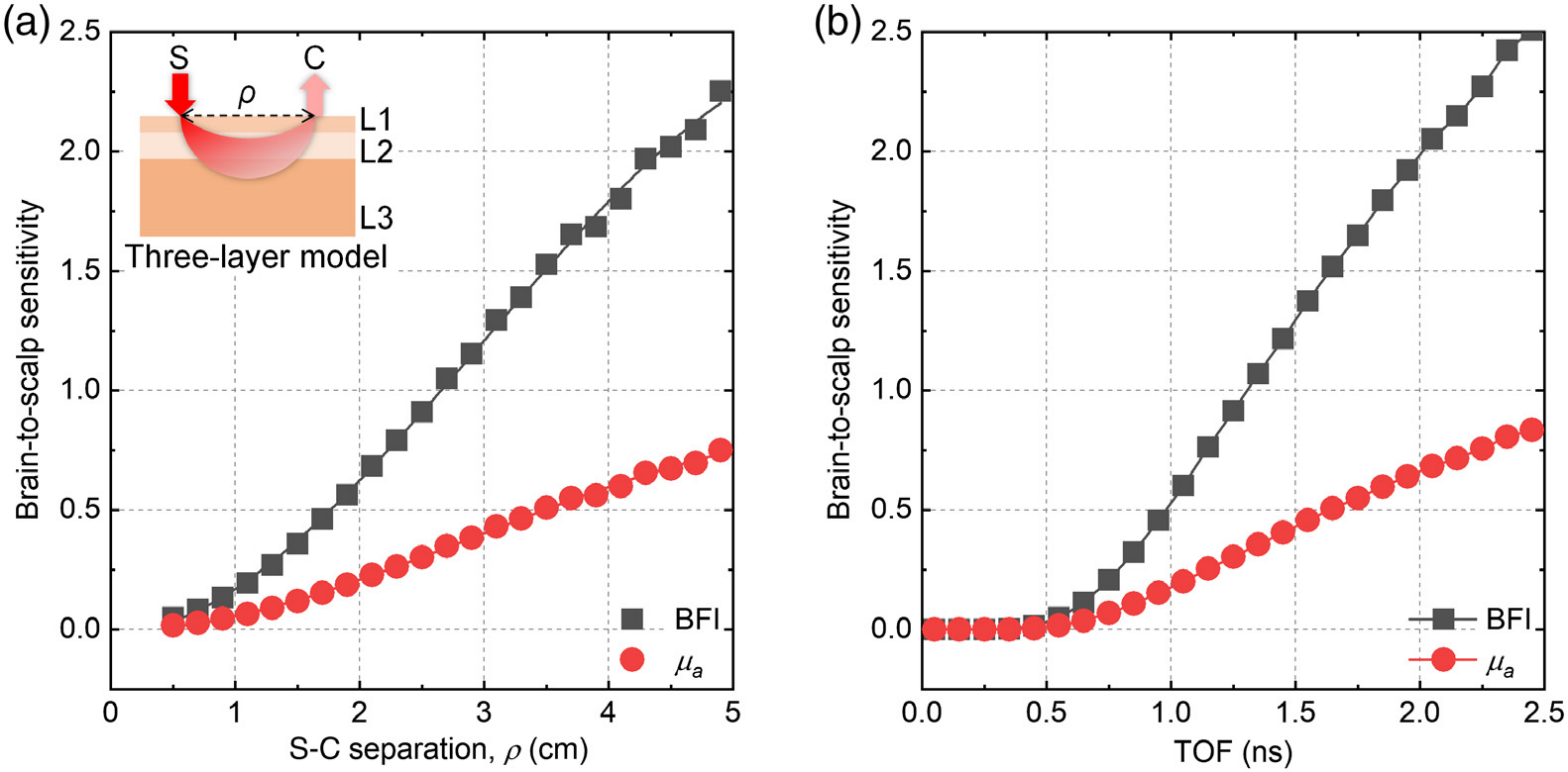
BFI is an intrinsically brain-specific signal. BFI outperforms light absorption ($\mu_{a}$) in terms of brain-to-scalp sensitivity (i.e., brain specificity) versus (a) S–C separation and (b) TOF. The brain-to-scalp sensitivities were simulated using a previously described Monte Carlo method^36^ with a three-layer model,^37^ where the BFI and $\mu_{a}$ ratios of brain to scalp were set at 6 and 2, respectively.^38^ Inset of (a): S, source; C, collector; ρ, S–C separation; L1, L2, and L3 represent scalp, skull, and brain layers, respectively, with thicknesses of 4.5, 7.5, and 88 mm,^37^ respectively, and commonly used optical properties.^39^ Note that the deviations of brain-to-scalp sensitivities at large (a) S–C separation and (b) TOF are caused by low collected photon counts in the Monte Carlo simulation.

In the late 1980s, soft matter physicists characterized the temporal fluctuations of diffusely scattered light under the rubric of diffusing wave spectroscopy (DWS).^20^ This approach was later applied to measure the biological blood flow index (BFI) under the name DCS in the mid-1990s (Fig. 1).^21^ Though diffuse correlation functions provide coarse information about long and short paths,^20^^,^^22^ today DCS remains essentially a CW technique,^3^^,^^23^ with rare exceptions.^10^^,^^24^ Yet, as in NIRS, the potential benefits of time-resolved detection for DWS and DCS were appreciated early. For instance, nonlinear gating approaches could resolve intensity fluctuations according to time-of-flight (TOF),^25^ yet these early efforts were inefficient, and thus infeasible, in biological tissue. The early 1990s saw the development of an interferometric approach for measuring backscattered light from tissue, using light coherence to temporally gate or filter detected light.^26^ This new approach was called optical coherence tomography (OCT). The analogy between direct TOF detection and coherence gating of OCT was appreciated, and the literature is peppered with examples of a coherence gate aiding measurements of multiply scattered light.^27^^–^^29^ Yet these early implementations essentially replicated the measurement geometry of OCT, with a very narrow coherence gate and a single detector, resulting in excessive loss of potentially useful scattered light signal. Though the efforts yielded useful insight into the transition between single and diffusive dynamic scattering, they were not applied *in vivo*. Swept source approaches, inspired by Fourier domain OCT,^30^ for investigating colloidal suspensions were implemented but were either never applied *in vivo*^31^ or had limited speed and longevity.^32^^,^^33^ Meanwhile, *in vivo* CW DCS began to show potential in monitoring the human brain^8^ and became the method of choice for clinical studies.^3^^,^^34^^,^^35^ Later, CLFs were found to theoretically provide ≥3 times more brain-specificity than absorption measured by NIRS at a given source–collector (S–C) separation or TOF (Fig. 2).^22^^,^^38^ Yet, for many years, this potential advantage was tempered by the inherently low-light throughput of conventional DCS/DWS, making it impractical to achieve such a high brain specificity in adults.^3^^,^^23^

Over the past 5 years, motivated in part by the successes of DCS, several groups have independently investigated interferometric detection for diffusely scattered light in living biological tissue.^12^^–^^15^^,^^36^^,^^39^^–^^43^ This area of investigation remains active. Already, several clear and tangible advantages of the interferometric approach have emerged. First, interferometric detection provides comparable or better performance than photon counting with a dramatically reduced cost per pixel.^12^^,^^15^^,^^36^^,^^42^ Second, modifying light temporal coherence is a powerful and flexible way to achieve TOF resolution or discrimination in an interferometric setup,^39^^–^^41^^,^^43^ removing ambiguities to accurate interpretation of signals. Third, as a consequence, interferometric detection has great potential for blood flow fluctuation signals,^36^ whereas conventional DCS is constrained by high cost and low throughput.^44^

In this work, we discuss core principles of interferometric detection, including the concept of heterodyne gain, signal-to-noise ratios (SNRs) of heterodyne and homodyne detection, choice of reference power, coherence gating, TOF-resolved detection, and 2D sensor approaches. Then we describe remaining issues to be understood and addressed, such as mitigating motion artifact, further improving the SNR and brain specificity, and reducing the light source cost.

## 2 Core Principles

### 2.1 Heterodyne Gain

In iDWS, heterodyne gain enables inexpensive sensors to measure weak sample light levels. Here we adopt the definition of “heterodyne” from dynamic light scattering,^45^ which differs from the definition in optical communications. In heterodyne DCS/DWS methods, a strong reference light field $E_{R}$ is interfered with a weak diffuse (sample) light field(s) $E_{S}(t)$:

P(t)∝|ER+ES(t)|2=|ER|2+|ES(t)|2+2 Re{ER*·ES(t)}, (1)

where P(t) is the measured power; $P_{S}(t)\propto {\left|E_{S}(t)\right|}^{2}$ is the homodyne sample power, which fluctuates over time due to sample dynamics; $P_{R}\propto {\left|E_{R}\right|}^{2}$ is the reference power, which is assumed to be constant; and 2 Re{ER*·ES(t)} is the heterodyne term, which includes the in-phase component of $E_{S}(t)$. The time-averaged reference and sample powers are ${\overset{¯}{P}}_{R}=P_{R}$ and ${\overset{¯}{P}}_{S}={\left\langle P_{S}(t)\right\rangle }_{t}$, respectively.

For a simple signal comparison between homodyne and heterodyne detection, we assume that (1) all powers are given in units of photoelectrons per second; (2) single detector/pixel for single speckle, indicating homodyne coherence factor^3^ β and heterodyne mutual coherence degree^12^ $\overset{¯}{\gamma }$ are both equal to 1; and (3) the detector exposure time T is much shorter than the decorrelation time of $E_{S}(t)$, $\tau_{c}$ (i.e., $T\ll \tau_{c}$), ensuring no decorrelation during the exposure time.^15^ Thus the heterodyne gain is the heterodyne to homodyne signal ratio of $2{\overset{¯}{P}}_{R}/{\overset{¯}{P}}_{S}$ (see Table 1).

**Table 1:** Signal and SNR for heterodyne and homodyne detection.

	Homodyne	Heterodyne
Signal	$({\overset{¯}{P}}_{S}T{)}^{2}$	$2{\overset{¯}{P}}_{S}{\overset{¯}{P}}_{R}T^{2}$
Heterodyne gain	$2{\overset{¯}{P}}_{R}/{\overset{¯}{P}}_{S}$	
$\sigma_{\text{shot}}^{2}$	${\overset{¯}{P}}_{S}T$	${\overset{¯}{P}}_{R}T$
${\mathrm{SNR}}_{\text{shot}}$	${\overset{¯}{P}}_{S}T$	$2{\overset{¯}{P}}_{S}T$
$\sigma_{\mathrm{tot}}^{2}$	${\overset{¯}{P}}_{S}T+\sigma_{\mathrm{cam}}^{2}$	${\overset{¯}{P}}_{R}T+\sigma_{\mathrm{cam}}^{2}$
${\mathrm{SNR}}_{\mathrm{tot}}$	$\ll {\overset{¯}{P}}_{S}T$	$∼2{\overset{¯}{P}}_{S}T$

$\sigma_{\text{shot}}^{2}$: shot noise variance; $\sigma_{\mathrm{tot}}^{2}$: total noise variance; $\sigma_{\mathrm{cam}}^{2}$: camera/detector noise variance; ${\mathrm{SNR}}_{\text{shot}}$: signal-to-shot-noise ratio; and ${\mathrm{SNR}}_{\mathrm{tot}}$: signal-to-total-noise ratio, where ${\overset{¯}{P}}_{R}T\gg \sigma_{\mathrm{cam}}^{2}\gg {\overset{¯}{P}}_{S}T$ for low sample power, high reference power, and noisy camera/detector.

**Fig. 3:**
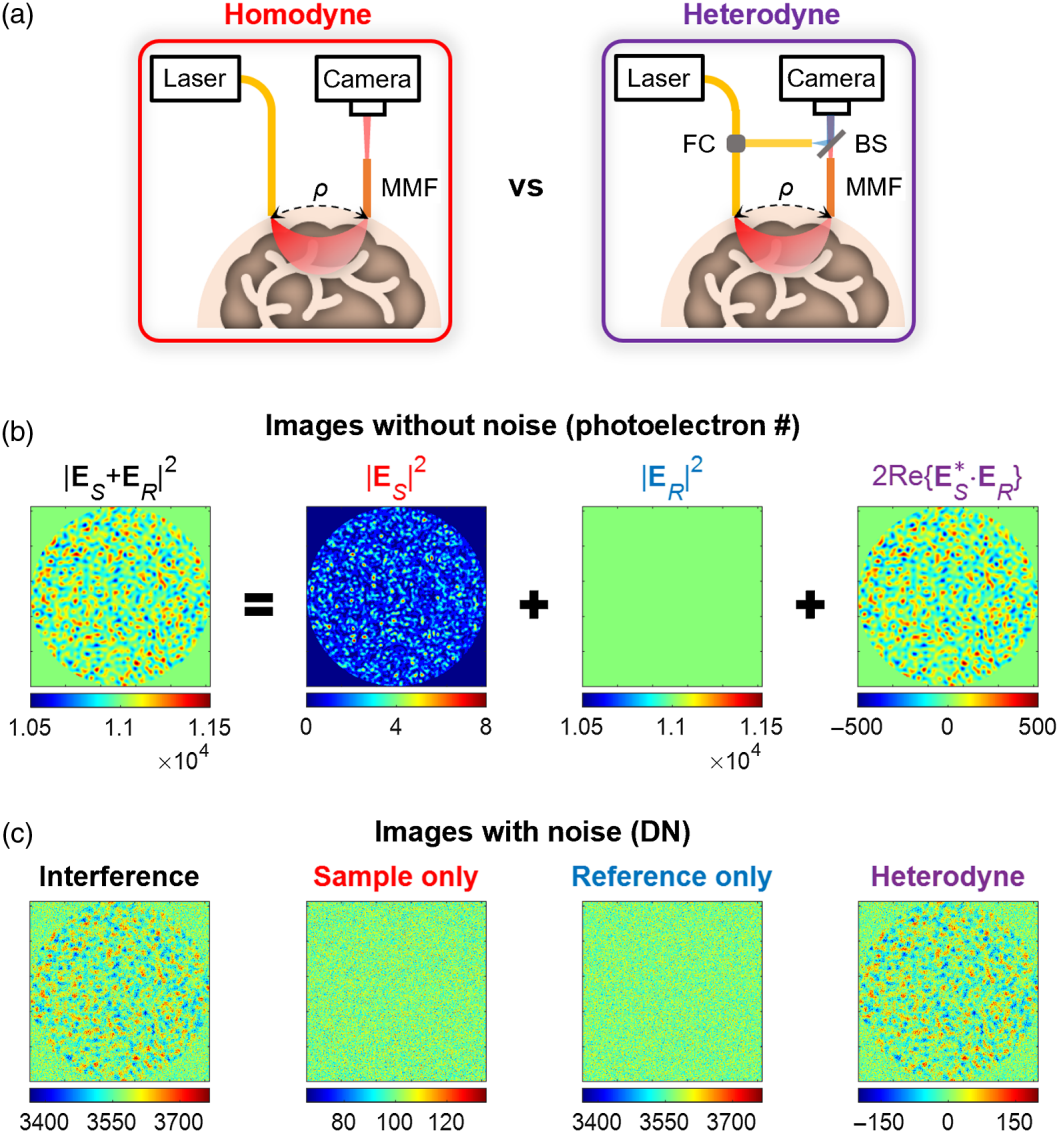
Strong reference light boosts weak sample signal in heterodyne detection. (a) Comparison between homodyne and heterodyne setups, with MMF and 2D camera collecting and detecting diffuse light, respectively. MMF, multimode fiber; FC, fiber coupler; BS, beamsplitter; ρ, S–C separation. (b) Simulated images of multimode interference between strong reference light, with uniform intensity pattern, and a weak sample light, with MMF speckle pattern. (c) Simulated 12-bit images with additive shot noise and camera noise under real measurement conditions, corresponding to images in (b). One digital number level is assumed to be ∼3 photoelectrons. The MMF speckle pattern is dominated by camera noise in the homodyne approach (sample only), but it is clearly visualized in the shot noise-limited heterodyne image.

Heterodyne gain or amplification is particularly beneficial for lower sample powers (Fig. 3). With an appropriate detector, we can achieve ${\overset{¯}{P}}_{R}/{\overset{¯}{P}}_{S}\gg 1$. Boosting the sample signal in this manner has three important advantages. First, it enables digitizing the signal of interest with more bits and thus higher fidelity [Fig. 3(b)]. Second, it boosts the associated signal and shot noise above the detector noise [Fig. 3(c)], so it is possible to reach the shot noise limit, as described in the next section. Third, it entails measuring the sample field, or at least its in-phase part, as opposed to the sample power. Thus the heterodyne method is sensitive to sample intensity and phase fluctuations, whereas the homodyne method is just sensitive to intensity fluctuations.^46^

### 2.2 Shot-Noise Limited SNR

Though heterodyne gain is a useful concept, it does not account for noise. For instance, a large reference power increases both the signal and the shot noise. For this reason, SNR is a more appropriate metric to compare detection approaches. First, we consider a noiseless detector in which the only noise source is shot noise ($\sigma_{\text{shot}}^{2}$). If ${\overset{¯}{P}}_{R}T\gg {\overset{¯}{P}}_{S}T$ for weak sample light, we find that ${\mathrm{SNR}}_{\text{shot}}=2{\overset{¯}{P}}_{S}T$ for heterodyne detection (Table 1), double that of homodyne detection. The factor of 2 in ${\mathrm{SNR}}_{\text{shot}}$ arises from the heterodyne signal term in Eq. (1).

Next, we consider detector or camera noise (i.e., total noise given by $\sigma_{\mathrm{tot}}^{2}=\sigma_{\text{shot}}^{2}+\sigma_{\mathrm{cam}}^{2}$). If ${\overset{¯}{P}}_{R}T\gg \sigma_{\mathrm{cam}}^{2}\gg {\overset{¯}{P}}_{S}T$ for weak sample light detected by a noisy sensor, we find that ${\mathrm{SNR}}_{\mathrm{tot}}\approx 2{\overset{¯}{P}}_{S}T$ is still obtained for heterodyne detection (Table 1), whereas ${\mathrm{SNR}}_{\mathrm{tot}}\ll {\overset{¯}{P}}_{S}T$ for homodyne detection, much lower than that of case 1. This is because heterodyne detection can achieve the shot noise limit even for a noisy camera/detector (i.e., $\sigma_{\text{shot}}^{2}\approx {\overset{¯}{P}}_{R}T\gg \sigma_{\mathrm{cam}}^{2}$), enabling us to “see” a small sample signal ${\overset{¯}{P}}_{S}$, which is otherwise buried in the camera noise [compare “heterodyne” and “sample only” in Fig. 3(c)].

### 2.3 Choice of Reference Power

The above considerations suggest that a reference count that is much larger than the sample count is beneficial. This ensures that the homodyne autocorrelation term is much smaller than the heterodyne autocorrelation term, which simplifies the interpretation of data. An ideal reference power should enable all pixels to reach the shot noise limit^12^^,^^15^^,^^36^ but, obviously, not cause the detector to saturate. A high reference power also places stringent requirements on stability so as not to induce additional spurious decorrelation dynamics from reference fluctuations. We have not observed excess noise that would preclude high reference powers in our experiments^36^ with isolated and temperature-controlled DFB and DBR lasers. Others have proposed signal processing strategies to deal with noise from mode hopping.^47^

Despite the benefits of a large reference count, several works have operated in a regime in which the reference count is comparable to the sample count.^48^ Whether this is a necessary consequence of using detectors with a low saturation level at short S–C separation, or whether there are actually advantages to working in this regime, remains unclear. Early work suggested the choice of an optimal reference power approximately equal to the sample power for measuring low-correlation levels,^49^ but a high reference power was later shown to be optimal for large S–C separations over much of the autocorrelation decay;^42^ it was demonstrated that relative variability of fitted BFIs monotonically decreased by up to 80% for a reference to total power ratio, $\alpha ={\overset{¯}{P}}_{R}/({\overset{¯}{P}}_{R}+{\overset{¯}{P}}_{S})$, increasing from 0 to ∼1. Data analysis in the case of comparable reference and sample powers can be handled by a modified Siegert relationship that includes a static term,^12^^,^^42^^,^^46^^,^^48^ which is expressed as

g2(τ)=1+2γ¯2α(1−α)Re{g1(τ)}+β(1−α)2|g1(τ)|2, (2)

where $g_{2}(\tau )$ and $g_{1}(\tau )$ are the normalized intensity and field autocorrelations, respectively, α is the reference to total power ratio, $\overset{¯}{\gamma }$ is the heterodyne mutual coherence degree,^12^ β is the homodyne coherence factor, and the standard Siegert relation of $g_{2}(\tau )=1+\beta {\left|g_{1}(\tau )\right|}^{2}$ is obtained by setting α=0. Note that the middle term of Eq. (2) is the heterodyne term of iDWS.^12^ In the case that $g_{1}(\tau )$ is real and ${\overset{¯}{\gamma }}^{2}=\beta$, Eq. (2) reduces to more common forms.^42^

### 2.4 Coherence Gating

**Fig. 4:**
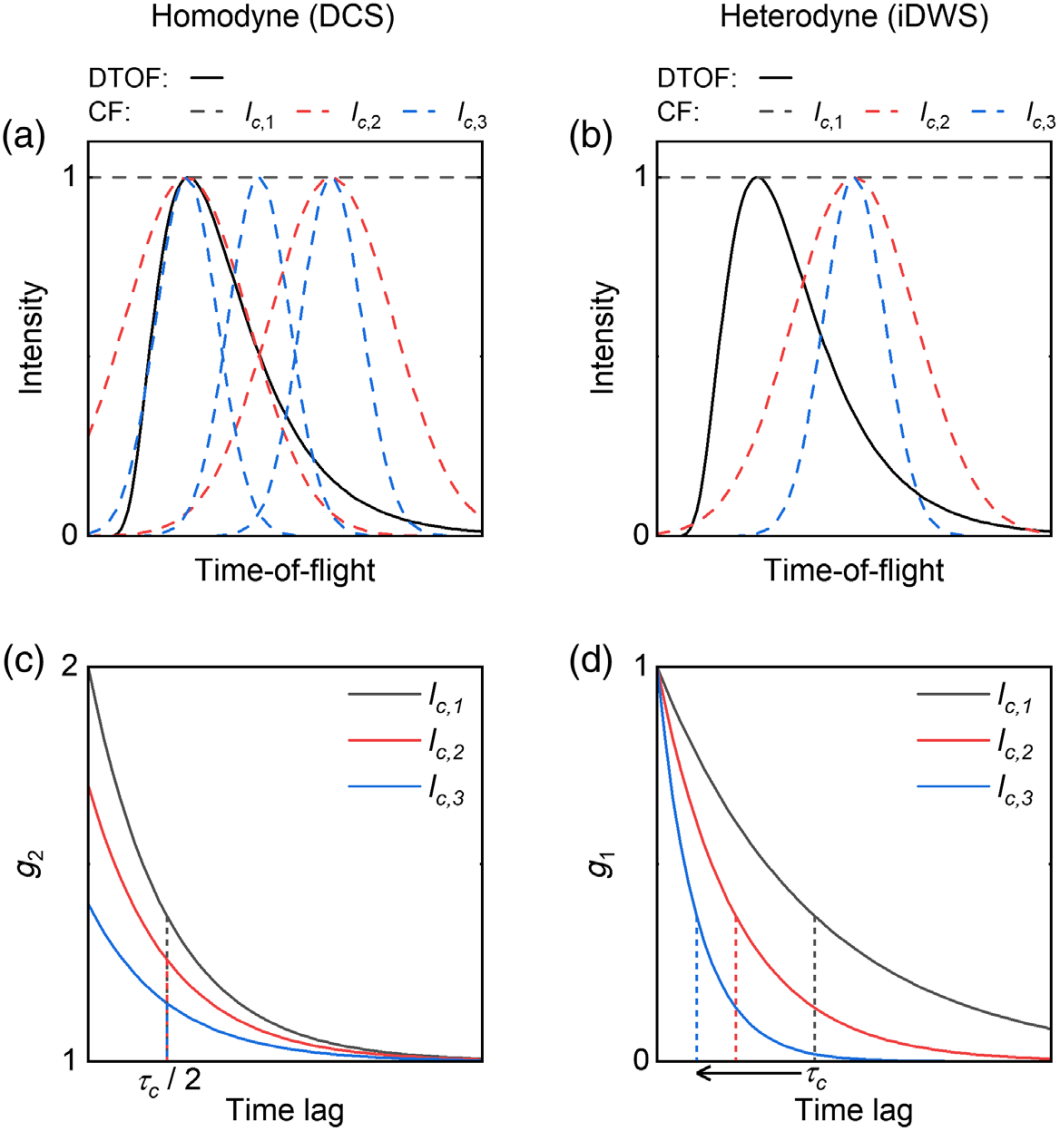
Source coherence affects homodyne and heterodyne techniques differently. Photon DTOFs, with coherence lengths from $l_{c,1}$ to $l_{c,3}$ for (a) homodyne DCS and (b) heterodyne iDWS. DTOF, distribution of times-of-flight; CF, coherence function; and $l_{c,1-3}$, coherence lengths of source light. (c) Reduced coherence decreases $g_{2}$ amplitude (β) for DCS. (d) Reduced coherence creates a TOF filter, affecting decorrelation time but not the $g_{1}$ amplitude for iDWS. $g_{2}$, normalized intensity autocorrelation; $g_{1}$, normalized field autocorrelation; and $\tau_{c}$, 1/e decorrelation time of $g_{1}$ (i.e., $\tau_{c}/2$ for $g_{2}$).

In homodyne DCS, all optical paths must be coherent with each other to maximize the available fluctuation signal (i.e., β of intensity autocorrelation). Reduced coherence superposes independent speckles and effectively reduces β [Figs. 4(a) and 4(c)]. Low coherence is undesirable in CW DCS and is unavoidable in time-domain DCS.^10^^,^^50^ In iDWS with a large heterodyne gain, the predominant signal is formed by the interference of sample paths with the reference path. A low-coherence light source can, therefore, create a TOF filter, attenuating paths that do not fall within the coherence function [Fig. 4(b)].^51^ TOF filtering reduces the height of $G_{1}$, but is beneficial, on balance, as superficial paths can be preferentially attenuated.^52^ Thus coherence reduction is a simple approach to achieving TOF filtering or discrimination, which can attenuate scalp photons to enhance brain sensitivity at small S–C separations [Fig. 4(d)].^39^

### 2.5 TOF-Resolved Interferometric Diffuse Optics

**Fig. 5:**
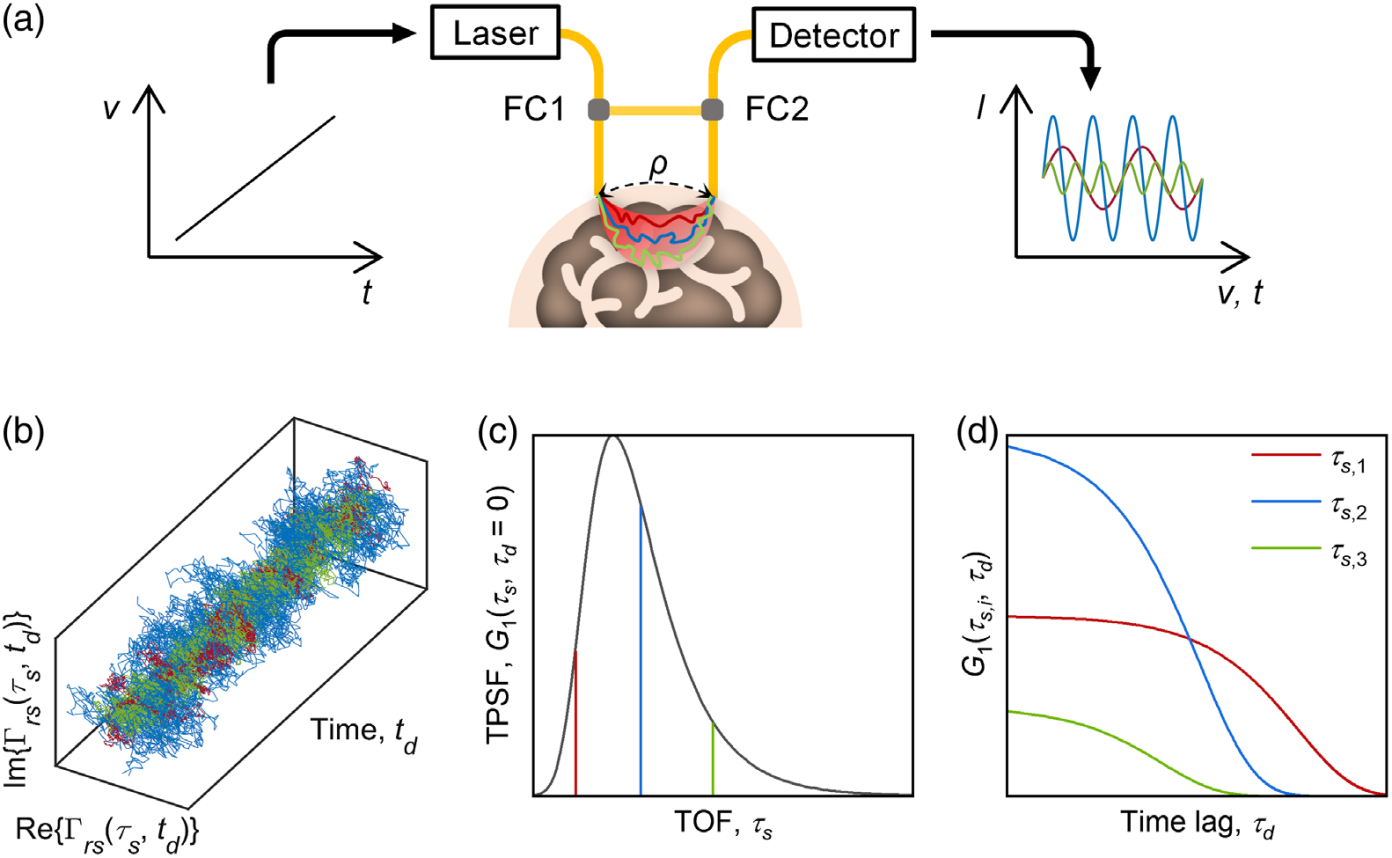
iNIRS measures intensity and dynamics with TOF resolution (a) by encoding TOF information in interference spectra acquired by frequency tuning of a laser source. FC1 and FC2 indicate single-mode fiber couplers. (b) Temporal fluctuations of the mutual coherence function $\Gamma_{rs}(\tau_{s},t_{d})$ obtained by inverse Fourier transformation of interference spectra. The autocorrelation of $\Gamma_{rs}(\tau_{s},t_{d})$ yields $G_{1}(\tau_{s},\tau_{d})$, which encapsulates both the (c) TPSF and (d) TOF-resolved field autocorrelations. $G_{1}(\tau_{s},\tau_{d})$ represents an information-rich two-dimensional data set from which optical properties and BFI can be derived. Red, blue, and green denote short, medium, and long TOFs in (a)–(d), respectively.

Going beyond mere TOF discrimination, TOF resolution aims for a full temporal point spread function (TPSF)^53^ and concordant TOF-resolved dynamics.^10^^,^^24^ iNIRS is an interferometric diffuse optical approach that measures superposed interference spectra, which are Fourier transformed to yield TOF-resolved complex optical field time courses, from which TOF-resolved intensity and field autocorrelations are determined (Fig. 5). iNIRS can separate static and dynamic components in the TPSF,^41^ providing rich information about multiple scattered light paths. TPSFs provide optical properties, which facilitate recovering absolute BFI.^3^^,^^23^^,^^35^ Admittedly, any technique providing TOF-resolved CLFs^10^^,^^40^^,^^51^ can also measure TPSFs and, in principle, with sufficient TOF resolution, quantify optical properties. However, unlike TD-DCS, iNIRS does not suffer from the tradeoff between coherence and TOF resolution^54^ and can achieve TOF resolutions of <25 picoseconds^41^ with a high dynamic range through spectral shaping.^54^ The major drawback of iNIRS is its intrinsically low-light throughput, resulting from the high cost of channels digitized at hundreds of MHz. As argued elsewhere, TOF-discrimination is compatible with highly parallel detection by less expensive sensor arrays.

### 2.6 2D Camera Interferometric Approaches

Recently, interferometric diffuse optical approaches have been adapted for low frame rate 2D cameras. Conventional iDWS uses a fast camera with a short exposure time T relative to the decorrelation time $\tau_{c}$.^12^ MiDWS relaxes this requirement, instead probing the autocorrelation indirectly by varying exposure time of a 2D camera with low frame rate.^15^ Other 2D approaches probe multiple heterodyne frequencies^13^ or even use a single exposure and measure the sample power separately.^14^ 2D approaches can theoretically achieve orders of magnitude lower cost per speckle than iDWS due to the massive number of available pixels ($10^{5}$ to $10^{7}$) on 2D cameras. In benefitting from 2D cameras, these approaches are akin to SCOS/DSCA (diffuse speckle contrast analysis), which measures BFI from homodyne speckle contrast(s).^11^^,^^55^^–^^58^ MiDWS and SCOS/DSCA are compared for human brain BFI measurements in Table 2.

**Table 2:** Comparisons of key features between SCOS/DSCA and MiDWS/iSVS for the human brain.

	SCOS/DSCA	MiDWS/iSVS
Relationship to autocorrelation	$\kappa^{2}=\frac{2\beta }{T}\int_{0}^{T}{\left|g_{1}(\tau )\right|}^{2}(1-\frac{\tau }{T})d\tau$	$\langle U_{\mathrm{AC}}^{2}\rangle \propto T\int_{0}^{T}g_{1}(\tau )(1-\frac{\tau }{T})d\tau$
Sensitivity to camera noise	High	Low
Single exposure measurement	Yes	Possible, with known ${\overset{¯}{P}}_{S}$^14^
Practical brain specificity	Low ($T\gg \tau_{c}$)	High ($T\ll \tau_{c}$)
To use short exposures	Yes, only with low $\sigma_{\mathrm{cam}}^{2}$	Yes
Maximum S–C separation	2.5 cm (T=2 ms)^57^	3 cm ($T_{\min}\approx 0.05\;\mathrm{ms}$)^15^

κ, speckle contrast^55^ and $\left\langle U_{\mathrm{AC}}^{2}\right\rangle$, mean square of the heterodyne signal.^15^

Is it possible to obtain TOF information while benefitting from the scalability of 2D interferometric approaches? A brute force approach that borrows concepts from iNIRS and iDWS is to employ a high-speed and expensive camera for parallelization of iNIRS.^59^ However, to achieve sufficient sampling of the interference spectrum for reconstruction of a full TPSF^41^ with this approach, even at frame rates of MHz, maximal sweep rates are, at best, in the tens of kHz range. An alternative strategy is to forego TOF resolution in favor TOF discrimination,^39^ which relaxes the frame rate requirements and is (in theory, at least) compatible with highly parallel multiexposure approaches.

## 3 Limitations, Questions, and Issues to be Solved

Notwithstanding their potential, interferometric diffuse optical techniques come with unique limitations that must be understood and addressed.

### 3.1 Motion Artifacts

Interferometric detection, which depends on fluctuations of both light intensity and phase, is more susceptible to motion artifacts than conventional intensity-based DCS/DWS. Moreover, many iDWS systems collect light with MMFs, the motion or vibration of which could induce dynamics beyond those intrinsic to the sample. We generally find that iDWS measurements are not that susceptible to moderate motion of a few meter collection MMF, given that the motional decorrelation dynamics are much slower than intrinsic sample decorrelation dynamics.^36^ To further mitigate MMF motion artifacts, an MMF bundle with a smaller core size and NA could be employed,^60^ as suggested by related work.^14^ Another issue for noncontact interferometric detection^41^^,^^43^ is axial motion between the subject and probe, which causes Doppler phase drifts resulting in overestimated sample dynamics. The iNIRS method solves this issue by measuring TOF-resolved complex optical fields, from which the Doppler shift can be quantified from static light at short TOFs, and such artifacts can be corrected.^41^

### 3.2 SNR or Brain Specificity

Even with recent advances, state-of-the-art iDWS has not yet reached the light throughput of widely used CW-NIRS, and measurements at large S–C separations (≥4  cm), at late TOFs, or in regions with overlying hair remain challenging.^36^ How do we further improve SNR by additional 1 to 2 orders-of-magnitude and better achieve theoretical advantages of BFI for brain specificity (Fig. 2)? Here we describe three potential solutions, not mutually exclusive, that are being investigated: first, enhancing SNR by further increasing the speckle number, based on parallelizing multiple cameras for iDWS/MiDWS, is a straightforward way to achieve larger S–C separation (i.e., higher brain specificity); second, combining iDWS with coherence gating, to filter out extracerebral photons with short TOFs at a small S–C separation, can improve both SNR (more cerebral photons) and brain specificity;^39^^,^^41^^,^^43^ third, the intrinsic advantages of SNR and high source power for DCS at 1064 nm can be carried over to interferometric detection,^61^^,^^62^ in which extremely costly 1064 nm photon counting detectors^63^ can be replaced by InGaAs cameras,^64^ which are less costly.

### 3.3 Light Source Cost

With detector technologies supported by economies of scale, we project that source cost will soon become a limiting factor in scaling interferometric diffuse optics. Benefiting from the high pixel counts of CMOS sensors, interferometric detection has reduced the cost of a single detected speckle by orders of magnitude^36^ compared with conventional multispeckle DCS/DWS.^44^ This trend is likely to continue. For instance, the 2D CMOS camera used in Fourier domain DCS^13^^,^^47^ only costs hundreds of dollars, which is dozens of times less expensive than the source. State-of-the-art iDWS^36^ has used similar light sources as state-of-the-art DCS/DWS^44^ to date. We believe that more systematic iDWS investigations with lower cost laser sources^65^ are warranted. Indeed, we have found that a lower temporal coherence, characteristic of lower cost sources, can actually be helpful to reducing the impact of unwanted system reflections in iDWS. Finally, although iDWS at 1064 nm can also reduce the laser source cost,^64^ InGaAs sensors unfortunately are expensive. Though not strictly a limitation, we project that lowering the light source cost, perhaps in conjunction with noise mitigation strategies,^47^ will be important to enabling new applications in the coming years.

## 4 Outlook

Functional NIRS developed from a single channel^4^ to high-density diffuse optical tomography (DOT)^16^ in two decades. Because photon counting does not easily scale, DCS/DWS has not seen similar progress, almost two decades since the first functional measurements.^8^ Technologies on the horizon such as SPAD arrays may eventually improve performance but have not yet done so.^66^^,^^67^ Interferometric detection presents one way to enhance both the SNR and performance-to-cost ratio to fully realize the advantages of BFI.^22^^,^^38^ Moreover, iDWS may scale to multiple detection channels with a lower cost than other technologies. Looking ahead, an interferometric diffuse optical tomography system could achieve more brain-specific functional mapping with same S–C pairs as conventional DOT, or higher spatial resolution (shorter S–C separation) with the same brain specificity as conventional DOT. With the rapid progress over the past five years, we predict that these exciting developments, which could benefit neurointensive care, functional neuroimaging, and brain–computer interfaces, are just on the horizon.
